# General Demographics and Behavioral Patterns of Visitors Using a Self-help Website for Identification of and Intervention in Alcoholism and Common Mental Disorders in Suriname: Descriptive Study

**DOI:** 10.2196/33793

**Published:** 2022-06-09

**Authors:** Raj Jadnanansing, Jack Dekker, Kajal Etwaroo, Rudi Dwarkasing, Vincent Lumsden, Robbert Bipat, Matthijs Blankers

**Affiliations:** 1 Psychiatrisch Centrum Suriname Paramaribo Suriname; 2 Faculty of Social Science Anton de Kom University of Suriname Paramaribo Suriname; 3 Vrije Universiteit Amsterdam Amsterdam Netherlands; 4 Arkin Amsterdam Netherlands; 5 Faculty of Medical Science Anton de Kom University of Suriname Paramaribo Suriname

**Keywords:** eHealth, mental health, alcohol use disorder, depression, anxiety, Facebook, alcohol disorder, alcohol, self-help, alcoholism, Suriname

## Abstract

**Background:**

Digital health applications have been shown to be an accepted means to provide mental health information and advice in various high- and middle-income countries. Started in 2015, ehealth.sr was the first website to offer preventive information, self-tests, and unguided digital self-help for depression, anxiety symptoms, and problematic alcohol use in Suriname, an upper middle-income country in South America.

**Objective:**

This study aimed to assess the general demographics and behavioral patterns of the visitors of ehealth.sr, as well as to evaluate different promotional channels to attract the target audience to the website.

**Methods:**

Data collection for this study took place between August 2015 and December 2020. Conventional promotion channels such as newspaper and radio advertisements as well as social media advertisements were used to attract users to the website. The number of visits and activity on the website was registered using Google analytics and the website’s internal activity log.

**Results:**

On average, about 115 unique visitors accessed the website per month. The average number of visits to the website increased notably when social media advertisement campaigns were conducted (266 per month in 2018) compared to when traditional advertisements campaigns through papers, radio, and television were used (34 per month in 2019). Of the 1908 new visitors, 1418 (74.32%) were female. On average, visitors accessed 2 (SD 0.3) pages of the website and a session lasted 2.6 (SD 0.9) minutes. The most popular pages for intervention on the website were those for the mood or anxiety screening (731/942, 77.6%) as opposed to those for alcohol screening (211/942, 22.4%). People aged <45 years (on average, 2.2 pages per session for 3.2 minutes) made more use of the website than people aged ≥45 years (on average, 1.7 pages per session for 2 minutes).

**Conclusions:**

Promotion via social media led to more visitors to the website than newspaper or radio advertisements. Younger age groups and females visited the website more often. The pages on preventive information and brief self-tests were visited more frequently than the self-help modules. In general, user adherence to the website in terms of the average session duration and number of viewed pages per session is low and is a key point of concern for the successful implementation of digital mental health websites.

## Introduction

In recent years, mental health has attracted the attention of digital technology [1] since it has the potential to transform the whole spectrum of mental health care by connecting patients, services, and health data in new ways [2]. Digital health (or eHealth as it is sometimes called) is a wide and varying concept that includes the use of communication technology for digital record keeping, web-based booking systems, web-based repeat prescriptions, and other innovative uses of technology for direct treatment [3]. The World Health Organization (WHO) defines eHealth technologies as the use of information and communication technologies for the evaluation and promotion of health [4].

The application of information and communication technologies to support national health care services is rapidly expanding and has become increasingly important globally [4]. Consequently, donor agencies and foundations invest in many digital health interventions in low- and middle-income countries (LMIC), aiming to provide a way to address major deficiencies in access to safe, effective, and affordable health services [5]. On average, nearly half the global population resides in countries where there is fewer than 1 psychiatrist per 200,000 inhabitants. This reality is reflected primarily in LMIC where, for example, up to 85% of patients with severe mental disorders do not receive treatment for their disorder [6].

The implementation of eHealth promises a number of potential benefits to the health system, including, but not limited to, increased efficiency in health care, improvement in quality, the reduction of the cost of care, and the improvement of health system governance [7]. With these aspects, the provision of health care extends beyond its conventional boundaries. In addition, the impact of eHealth is that it enables personalized mental health care throughout the health system. Furthermore, it makes mental health care available at home, work, and school and focuses on prevention, education, and self-management outside the boundaries of clinics and hospitals [4].

Due to the wide availability of information and communication technologies, LMIC have developed eHealth strategies to prevent and treat mental health disorders and substance use disorders. For instance, HDep (Help for Depression or Ayuda para depresión [ADep] in Spanish) is an open-access and free web-based, psychoeducational, and cognitive-behavioral mental health program created in Mexico with a single goal—to help recognize and prevent depression [8]. HDep has the potential to serve as a useful tool for educating people about depression in order to change negative thinking patterns and be a source of social support [8].

As a low-income country, Bangladesh received special attention in 2013 for its innovative use of eHealth, especially in a mental health setting [9]. A detailed probe of the health system concluded that Bangladesh has made enormous progress and currently has the longest life expectancy and lowest mortality rates of infants and children under 5 years in South Asia, despite spending less on health care than several neighboring countries. Much of this can be attributed to the contribution of a web-based electronic health record and mobile Health innovations [10].

The advent of eHealth could thus be a powerful strategy for the delivery of mental health services, especially in LMIC. In this paper, we focus on web-based mental health care in Suriname, an upper middle-income country. In Suriname, eHealth is made possible through the website [11] designed by Arkin Mental Health Care and the Center for Psychiatry in Suriname (PCS) and hosted by the PCS. Previous studies have found a huge gap in the treatment of mental disorders such as alcohol abuse and depression and anxiety disorders [12,13]. These studies identified a treatment gap in Suriname, a transitional country. This is the main reason why more attention has been given to a digital health intervention to address substance abuse and mental health disorders in Suriname 12,13. Currently, it is difficult for people to find specific websites on the internet and make the distinction in quality between different sites [14]. Targeted advertisement is frequently recommended for those seeking specific information and help [15].

The aim of this study was to assess the general demographics and behavioral patterns of visitors on a mental health website offering psychoeducational information, self-tests, and unguided self-help interventions developed in LMIC. In addition, we also investigated the best way we can attract the target audience to a mental health website for LMIC such as Suriname.

## Methods

### Setting

Suriname is a middle-income, Caribbean, and South American country with approximately 575,990 inhabitants. Web-based mental health for the Surinamese community is made possible through the eHealth.sr website [11] hosted by the PCS. This website was developed in collaboration with Arkin Mental Health Care in the context of a survey research project on the prevalence and treatment gap for substance use disorders, depression, and anxiety among the population of the Surinamese districts Nickerie and Paramaribo [12,13]. Respondents in this survey project who scored above cutoff points for alcohol abuse, depression, or anxiety were referred to the eHealth.sr website.

### eHealth.sr Website

The eHealth.sr website contains modules with psychoeducational information, self-screening tests, and structured self-help for problematic alcohol use, depression, and anxiety. The website is offered in the main language of Suriname, which is Dutch. Most inhabitants of Suriname have at least a basic understanding and reading ability in Dutch.

### Problematic Alcohol Use Modules

The psychoeducational information module on alcohol addresses the short- and long-term risks of drinking, including the risk of developing alcohol use disorder and what can be done to mitigate these risks.

The self-test module is a questionnaire with feedback and includes a 7-day timeline follow-back to assess alcohol use in the past week, the Alcohol Use Disorder Identification Test, an alcohol-problems screening instrument developed by the WHO [16], and the Readiness to Change Questionnaire–Drinking [17], which is based on the Transtheoretical Model of Change [18].

After completing the questionnaires, the participants will receive automated personalized normative feedback. The self-help module contains a fully automated digital alcohol self-help program, originally developed by Jellinek [19] and not supervised by a professional. This multisession program is based on cognitive behavioral therapy and motivational interviewing (MI) techniques. The original version of this self-help intervention has shown positive results on reductions in alcohol use [20].

In this self-help intervention, various options that enable the person seeking help to determine the advantages and disadvantages of both the use as well as quitting of alcohol are offered. The participant can also set targets for themselves, visit the forum, and keep a diary. The module is an MI-based program and provides help-seeking tips and an overview of the results achieved in the self-help program. The part for problematic alcohol use has a time limit of 6 weeks, and eventually, the program fidelity and its achieved goals are displayed [11]. The module also offers the possibility for users to chat with each other and share their experiences with one another. All users are asked to create an account using their email address and a password before they can use the self-help program.

### Depression and Anxiety Modules

The psychoeducational information module on depression and anxiety addresses the common symptoms of depression, stress, anxiety, and suicidal tendencies, as well as the prevention and treatment measures that can be taken to mitigate these symptoms.

The self-test module is a questionnaire with feedback and includes the Center for Epidemiologic Studies Depression (CES-D) scale [21] and the General Anxiety Disorder-7 (GAD-7) questionnaire [22]. The CES-D scale is a 20-item measure that rates the frequency of experiencing symptoms associated with depression, such as restless sleep, poor appetite, and feeling lonely, by respondents in the previous week. The GAD-7 questionnaire is a brief self-report scale that is used to identify probable cases of general anxiety disorder. It is reliable and shows good criterion, construct, factorial, and procedural validity [22]. After completing the questionnaires, the participants will receive automated personalized normative feedback.

The self-help module contains a fully automated digital alcohol self-help program based on problem-solving therapy (PST) and MI. It was inspired by the PST-MI Exercises of the Substance Use and Trauma Intervention (eSTRIVE) [23]. The findings of this study suggested that eSTRIVE appeared to be an effective brief intervention among adults presenting to emergency departments in South Africa [23].

Our self-help program offers PST-MI in 4 sessions. The participant will learn to recognize and handle problems as either unimportant or irrelevant, important but not resolvable, or important and resolvable. The goal for them is to regain control of their life, discover what is important to them, and learn how to conquer problems like nervousness, depression, or anxiety.

### Procedure and Data Collection

The website [11] was launched on August 1, 2015, prior to the population survey in Nickerie (which was held from August 15-27, 2015). Interest was generated in this period during the informative sessions with the doctors, district commissioners, police officers, and civil servants that helped during the survey.

At various occasions, promotion efforts were made to attract the target audience to the website by handing out eHealth flyers at regional health care centers and through the Facebook page and newspaper advertisements. The 2015-2016 population survey in Nickerie and Paramaribo took place from July 23 to August 17, 2016, during which all respondents were informed and handed a folder about the website [11]. During these months, extra nationwide promotion took place via radio interviews and newspaper advertisements. In August 2018, we created a Facebook page and used Facebook advertisements to attract the target audience to the website. Throughout the whole year in 2019, we advertised the existence of the website once a week in daily newspapers as well as on several occasions on television and radio programs. Accordingly, we used the data obtained in 2016 and 2019 to assess the effects of traditional media and the data obtained around August 2018 to assess the effect of promotion through social media.

Page views and visitors’ behavior were recorded anonymously using Google Analytics. The responses of the participants in the self-test were also recorded anonymously to give them automated normative feedback. “Users” refers to the number of unique visitors to the site, whereas “sessions” refers to the total number of visits to the site, which includes both new and repeat visits [24]. Data in the self-help program were not anonymous, and as no informed consent was sought from the self-help participants, we cannot report the data collected in the self-help programs. To present data on the use patterns of the website modules, we used descriptive statistics.

### Ethical Considerations

As no data were prospectively collected solely for the purpose of this study and no participants were subjected to research procedures, this study was deemed exempt from medical ethics approval. The website is a result of a larger study that was approved by the Ministry of Health of Suriname (CMWO201504). Due to the anonymity of the persons entering their data on the site, consent was not required. In addition, consent was not obtained to guarantee the anonymity of the participants, thus they were prevented from entering personal data on any occasion.

## Results

The average number of monthly visits to the eHealth website from 2015 to 2020 were 202 per month in 2015, 94 per month in 2016, 39 per month in 2017, 266 per month in 2018, 34 per month in 2019, and 59 per month in 2020. On average, about 115 unique visitors accessed the website per month. The value for 2015 represents only 5 months because the program started in August. We saw a relatively high monthly number of visits in 2015 and 2016 and then again in 2018, with a small peak in 2020. The high number of visits of 2015 and 2016 were probably due to the introduction of the website to individuals during the survey. In 2018, there was an active advertisement through the Facebook page of the institute. A systematic review recommended this type of advertising as being extremely successful [25]. The number of visits to the website increased notably when social media advertisement campaigns was conducted (with a peak of 2809 in August 2018) compared to when traditional advertisements campaigns through papers, radio, and television were used (34 per month in 2019). In 2020, COVID-19 limited in-person visits to the clinics because all care was downgraded to emergency cases only.

Table 1 shows the gender distribution of the website visits. More female than male visitors made use of the website—1418 of the 1908 (74%) new visitors were female. Table 2 represents the age distribution of the sessions. We observed that the number of sessions increased with age.

**Figure 1:**
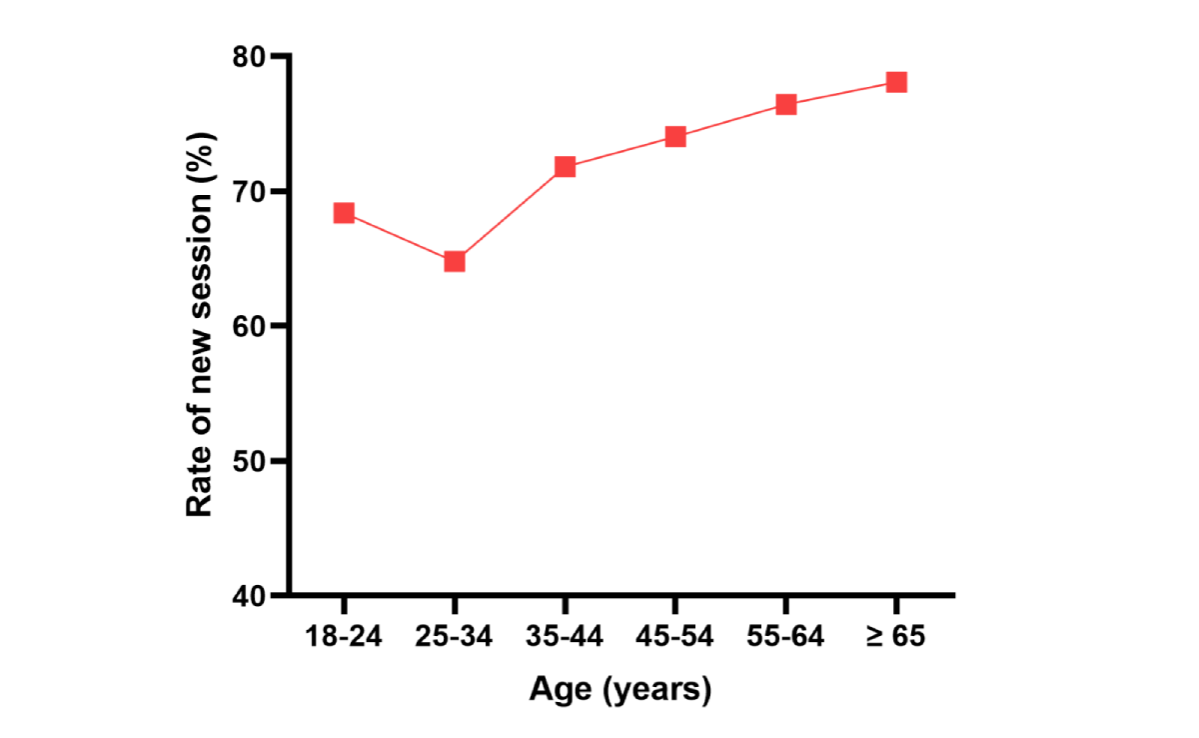
Rate of new sessions per age group.

**Figure 2:**
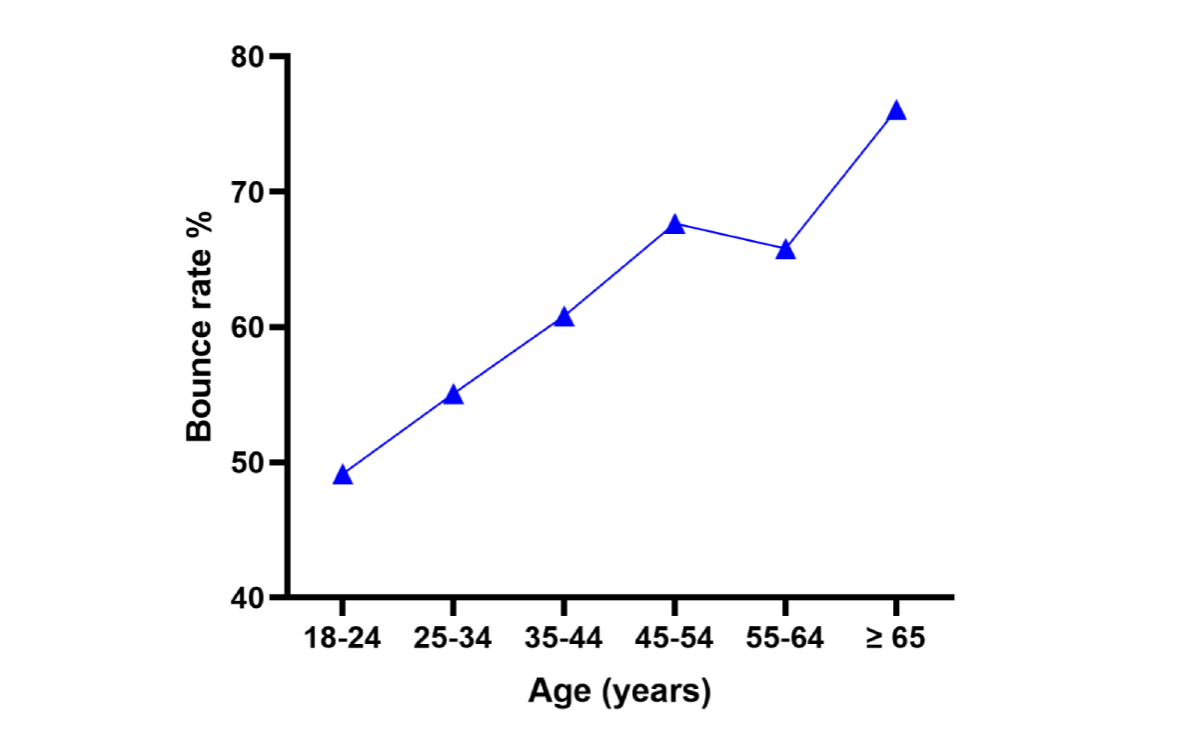
Bounce rate per age group.

Figure 1 shows the fraction of new sessions compared to the total sessions distributed by age, whereas Figure 2 shows the bounce rate (the percentage of sessions with only 1 page visited). On average, visitors accessed 2 (SD 0.3) pages of the website and a session lasted 2.6 (SD 0.9) minutes. The most popular pages on intervention modules on the website were those for the mood or anxiety screening (731/942, 77.6%) as opposed to those for alcohol screening (211/942, 22.4%). People aged <45 years (on average, 2.2 pages per session for 3.2 minutes) made more use of the website than people aged ≥45 years (on average, 1.7 pages per session for 2 minutes).

Table 3 shows the average time spent on the site. There were 2 peaks: the initial 10 seconds spent at the pages and sessions taking longer than 3 minutes. Thus, the majority of the participants decided within 10 seconds to quit or to continue for longer than 3 minutes.

Table 4 gives an overview of the numbers of participants starting the screening program for alcohol and mood or anxiety disorders and finishing the last page of the module. The number of participants finishing the modules for mood or anxiety disorders was more than those for possible problems with alcohol (odds ratio 2.137, 95% CI 1.583-2.888).

Table 5 presents the number of participants who were invited to enter the intervention for self-help for problem drinkers and mood or anxiety symptoms (eSTRIVE). The follow-up invitation was given 1 month after the initial invitation, while the participants were reminded after 1 week to finish the module. The number of participants of the mood or anxiety symptoms module (n=165) was notably higher than the number of participants of the problem drinking module (n=20). Overall, compared to the number of website visitors, the uptake of the self-help modules was low.

**Table 1:** Gender distribution of website visits.

Gender	New user (n=1908), n (%)	Session (n=2605), n (%)
Female	1418 (74.32)	1951 (74.89)
Male	490 (25.68)	654 (25.11)

**Table 2:** Session characteristics by age group distribution.

Age group (years)	Number of sessions (N=2528), n (%)	Pages viewed per session, mean (SD)	Average session length (s), mean (SD)
18-24	291 (11.51)	2.31 (0.4)	237.24 (92)
25-34	443 (17.52)	2.18 (0.3)	192.53 (75)
35-44	337 (13.33)	2.09 (0.3)	140.55 (55)
45-54	374 (14.79)	1.8 (0.3)	125.03 (49)
55-64	535 (21.16)	1.89 (0.3)	138.59 (54)
≥65	548 (21.68)	1.55 (0.2)	93.19 (36)

**Table 3:** The frequency of average time in seconds spent on the site.

Length of session (seconds)	Number of sessions (n=8973), n (%)	Number of pages displayed (n=14,914), n (%)
0-10	6888 (76.76)	7470 (50.09)
11-30	385 (4.29)	949 (6.36)
31-60	272 (3.03)	686 (4.6)
61-180	311 (3.47)	1017 (6.82)
181-600	642 (7.15)	2337 (15.67)
601-1800	421 (4.69)	1998 (13.4)
>1800	54 (0.6)	457 (3.06)

**Table 4:** The number of participants starting and finishing the screening program for alcohol and mood or anxiety disorders.

Screening	Participants entering the site (n=942), n (%)	Participants finishing the last page (n=521), n (%)
Alcohol	211 (22.3)	62 (11.9)
Mood or anxiety	731 (77.6)	459 (88.1)

**Table 5:** The number of participants invited to enter the intervention for self-help with suspected alcoholism and mood or anxiety disorders (eSTRIVE^a^).

Module			Invited		Reminded		Completed
**Alcohol self-help**							
	Baseline (n=20), n (%)	20 (100)		18 (90)		4 (20)	
	Follow-up (n=20), n (%)	20 (100)		20 (100)		1 (5)	
**Mood or anxiety disorders (eSTRIVE)**							
	Baseline (n=165), n (%)	165 (100)		154 (93.3)		19 (11.5)	
	Follow-up (n=159), n (%)	159 (100)		157 (98.7)		7 (4.4)	

^a^eSTRIVE: Exercises of the Substance Use and Trauma Intervention.

## Discussion

### Principal Findings

This study aimed to describe the general demographics and behavioral patterns of the visitors of an eHealth website provided by the PCS and the impact of the different forms of promotion on this site. We found that younger age groups were more likely to use the site for diagnostic and intervention purposes. There were more female than male visitors to the website. Furthermore, we observed that the eHealth module parts that were less intensive got more visitors than those that were more intensive since the launch in 2015. The number of visitors of the eSTRIVE module for depression were also higher than the module for problematic alcohol use. Another important finding is that whenever there was an active or intensive campaign in person or through social media, the number of visitors increased. Regular advertisement in newspapers and other nondigital media did not result in the same number of visits. Additionally, there was a small peak in 2020, which is probably ascribable to the measures taken with COVID-19, where clinics were closed and only used for emergency cases.

### Comparison With Prior Work

The decline of internet use for eHealth with age that we observed is very consistent with findings in high-income countries. A population-based survey from 2005-2007 in several European countries showed that the use of internet modules for general health was highest among people aged 18-25 years (70% to 80%) and declined progressively to under 20% among people aged >65 years [26]. Another survey in 2012 in the United States showed that the odds ratio for someone in the age group of 18-34 years who looked for health information on the internet to self-help was 3.51 (95% CI 1.66-7.44) compared to someone from the age group of >65 years [26]. In contrast to our findings, none of the abovementioned studies found a difference in gender use. However, a more recent Spanish study found that women used the modules more than men [27]. In accordance with our findings, the Spanish study also found that the modules for mood disorders were highest in use among the other modules [27].

Comparable studies in LMIC are not available. To our knowledge, this study is the first one analyzing these data in LMIC. The study shows that with proper advertisement it is possible to apply eHealth modules for mental disorders in LMIC. This is favorable since recent surveys have shown a significantly high treatment gap for alcohol abuse disorders [12] and depression and anxiety [13]. The program fully adheres to the WHO Mental Health Gap Action Program. First, it stimulates the potential patients to look for help. Second, it brings the patients closer to the availability of mental health professionals, and in this way also calls for the commitment of these professionals as stated in the goals of the WHO program.

Finally, we found that personal promotion and advertisements through social media resulted in the largest increase of visitors to the website. Traditional promotion through advertisements in the local newspapers and conventional media like radio and television did not contribute to a higher number of visits to the site. In general, peers and facilitators may increase the success rate of remote health programs [28], and this can be applied to promotion through social media [25].

### Limitations

A limitation of the program is that the promotion was initially in only 2 regions of the country, which could bias the number of visits to the website. However, more than 75% of the population resides in the capital, which minimizes the amount of expected bias. Another limitation is that the module is offered only in Dutch, thereby excluding those who do not use the language. Furthermore, the requirement of decent internet to complete the survey and then enter a possible treatment module limits the availability for the entire population. Due to the anonymous nature of the website, we were unable to assess the real-world impact of these limitations.

### Conclusion

In summary, we found that younger age groups and females visited the website more often. Furthermore, there was a success rate of at least 25% for both the alcohol abuse and depression or anxiety modules. Finally, we found that advertising through Facebook had the best effect on the use of these modules. We recommend that the use of an eHealth mental health intervention module is feasible to bridge part of the treatment gap in LMIC such as Suriname, if it is promoted adequately. Furthermore, to increase the use of the facility, it would be necessary to promote this kind of intervention among the older adults and men. We also recommend that the best way for promotion is through the personal involvement of doctors, nurses, and students. Advertisement through social media seems to work the best in these settings. Since the use of smartphones nowadays is more acceptable and widespread than desktop and laptop devices, it is recommended that future research develop a user-friendly app for smartphones.

## Abbreviations

CES-D: Center for Epidemiologic Studies Depression
eSTRIVE: Exercises of the Substance Use and Trauma Intervention
GAD-7: General Anxiety Disorder-7
LMIC: low- and middle-income countries
MI: motivational interviewing
PCS: Center for Psychiatry in Suriname
PST: problem-solving therapy
WHO: World Health Organization
